# The effect of probiotic supplementation on perceived stress and bowel function in healthy young adults: evidence from a randomized controlled trial in Makkah

**DOI:** 10.3389/fnut.2025.1717047

**Published:** 2026-01-06

**Authors:** Essra A. Noorwali, Abeer M. Aljaadi, Wafaa F. Abusudah, Fatmah A. Bakhdar, Dania H. Bin-Ali, Amani Alshinawi, Asma Bawazir, Raghad A. Mutlaq, Heba A. Maimany, Layan A. Barnawi, Bshayer Murshed, Bayan Aljared, Firas S. Azzeh

**Affiliations:** 1 Clinical Nutrition Department, Faculty of Applied Medical Sciences, Umm Al-Qura University, Makkah, Saudi Arabia; 2 Pharmacology and Toxicology Department, Faculty of Pharmacy, Umm Al-Qura University, Makkah, Saudi Arabia; 3 Department of Biology, Preparatory Year Program, Batterjee Medical College, Jeddah, Saudi Arabia

**Keywords:** probiotics, perceived stress, bowel function, gut–brain axis, randomized controlled trial, Saudi Arabia

## Abstract

**Background:**

Young adults experience high, persistent stress due to academic, social, and financial pressures. *Lactobacillus rhamnosus* GG (LGG) may reduce stress via the gut–brain axis, yet evidence from Middle Eastern populations is limited. We tested whether LGG lowers perceived stress in Saudi young adults and improved bowel function.

**Methods:**

In this randomized controlled trial, healthy adult participants with moderate–high Perceived Stress Scale (PSS) scores received *LGG* (6 × 10^9^ CFU/day, capsule) for 30 days or no intervention. Validated PSS version, anthropometrics and bowel habits were assessed at baseline and endline. Analyses included paired, two-sample t tests, Wilcoxon rank-sum and multivariable linear regression adjusted for baseline PSS, age, sex, and BMI were conducted.

**Results:**

Sixty-six participants completed the trial (37 probiotic; 29 controls; mean age 21.97 ± 2.59 vs. 20.83 ± 1.91 years). Post-intervention, stress score reductions were significantly greater in the probiotic group than controls (*p* = 0.006). In sex-stratified analyses, males receiving probiotics showed larger reductions than male controls (*p* = 0.007), while no significant difference was observed among females (*p* = 0.341). Probiotic participants also reported lower post-intervention stress scores (14.81 ± 6.12 vs. 19.48 ± 5.91; *p* = 0.003) and a higher proportion classified as low stress (84.2% vs. 15.8%; *p* = 0.008). Adjusted models showed control participants had stress scores 3.79 points higher than probiotic recipients (95% CI 0.74–6.83; *p* = 0.016). No between-group differences were found in bowel movement frequency, consistency, or GI symptom improvement.

**Conclusion:**

A 30-day *LGG* course may reduce perceived stress—particularly in males—with a trend level effect observed in females without affecting bowel habits. Probiotics may be considered as an adjunct for stress management in high-risk young adult populations. Future larger, placebo-controlled, and longer-term trials are recommended to confirm these findings and explore underlying mechanisms.

**Clinical trial registration:**

This trial is registered at ClinicalTrials.gov (Identifier: NCT06464484; https://clinicaltrials.gov/study/NCT06464484).

## 1 Introduction

Stress is defined as “a state of worry or mental tension caused by a difficult situation” based on the World Health Organization (WHO) (1). According to the National Institute of Mental Health, stress may arise from a single event or multiple occurrences, including positive life changes, and it can manifest as either acute or chronic (2). Stress may stem from various sources such as occupational demands (3), personal life responsibilities and academic pressures. Stress is a universal experience and happens in response to perceived threats and challenges however; the way individuals manage and react to stress significantly influences their overall health and well-being (1). Physiological, psychological, and social responses occur when individuals are stressed. Common symptoms of stress can manifest in various ways. Physically, stress often presents as muscle tension, pain, excessive sweating, headaches, digestive issues, rapid heartbeat (4) and changes in appetite—either overeating or undereating (5). Socially, individuals may experience nervousness, agitation, irritability, or anger, along with decreased focus and diminished job performance. Psychologically, stress may lead to anxiety, difficulty sleeping, memory lapses, and trouble making decisions. It’s important to note that these are some of the most frequently observed signs, but other symptoms may also occur depending on the individual (6). High stress in individuals may lead to unhealthy behaviours such as smoking (7), alcohol abuse (8) and unhealthy eating (5). Studies have shown that high stress is associated with an increased risk of several diseases such as mental disorders (9), cardiovascular diseases (10), hypertension (11), digestive diseases (9) and obesity (12). Collectively, this body of evidence highlights that managing stress is essential not only for mental well-being but also for preventing a cascade of unhealthy behaviors and related chronic diseases.

Globally, the prevalence of stress in more than 300,000 participants across 131 countries is 35% with higher prevalence among females and high-income countries (13). The WHO reports that stress, depression, and anxiety result in the loss of 12 billion workdays annually worldwide, costing the global economy approximately $1 trillion (14). A recent systematic review and meta-analysis found that mental disorders are highly prevalent in the WHO Eastern Mediterranean Region, with depression, generalized anxiety disorder, and post-traumatic stress disorder being the most common (15). Stress is particularly prevalent among students in the medical field (16). A Saudi study reported that 68% of 938 residents were stressed and 84% of the residents considered the job environment stressful (17). Furthermore, high stress was reported in Saudi healthcare providers (18), among nurses (19), healthcare university students (20) and during COVID-19 (21). A systematic review for over two decades reported stress ranging from 30 to 90% among medical trainees in Saudi Arabia (22). This highlights the need for effective stress management and to develop accessible and practical methods for reducing stress across different populations.

The gut microbiota plays a crucial role in regulating brain function and behavior. This regulation occurs through the gut-brain axis, a multifaceted communication network that includes neural, hormonal, and immune signaling pathways. Through these mechanisms, gut microbes can influence brain activity, emotional states, cognitive processes, and may contribute to the onset or progression of neuropsychiatric conditions (23). Studies suggest that probiotics may help prevent and manage diseases such as diabetes, heart disease, anaemia, infections, cancer and depression (24). A promising strategy for managing stress responses is through modulation of the gut–brain axis, where the gut microbiota serves as a critical regulator of stress-related processes. Emerging evidence suggests that probiotics may offer a simple and accessible strategy to modulate this connection and potentially alleviate stress-related symptoms in rodents (25), depressed individuals under stress conditions (26), individuals with anxiety (27) and in stressed healthy adults (28, 29). Saudi Arabia, classified as a high-income nation by the World Bank (30), is witnessing a rising prevalence of stress—highlighting the urgent need to investigate and implement effective therapeutic strategies to support population mental health. However, there remains a lack of sufficient studies focused specifically on the Saudi population. To date, no randomized controlled trial (RCT) has been conducted among Saudis (29); therefore, the aim of this study is to investigate the effects of probiotics in healthy, stressed Saudi young adults using a randomized controlled trial design on perceived stress and bowel function.

## 2 Materials and methods

### 2.1 Study design and participants

A RCT was conducted (Supplementary Figure S1) among young adults; mainly university students from Umm Al-Qura University (UQU), Makkah, Saudi Arabia. Ethical approval was obtained from the Biomedical Research Ethics Committee of UQU No. HAPO-02-K-012-2023-01-1414. This trial was registered at www.clinicaltrials.gov as NCT06464484 on 2024-06-20. This study was carried out in accordance with the principles of the Helsinki Declaration. Participants were recruited via email and several social media platforms from 26 January 2023 to 31 March 2024. Participants attended 2 visits at the nutrition clinic at the Faculty of Applied Medical Sciences, UQU.

### 2.2 Inclusion/exclusion criteria

#### 2.2.1 Inclusion criteria

Inclusion and exclusion criteria were selected based on a previous study (29) and on factors that may influence stress and the gut microbiota (31). No study has been conducted in healthy Saudi adults assessing the effects of probiotics on stress and bowel movements (29); therefore, the eligibility to participate in the study was Saudi young adults aged ≥ 18 years who have moderate or high levels of stress as was assessed during the first visit by the Perceived Stress Scale (PSS) (32). The study included both healthy males and females.

#### 2.2.2 Exclusion criteria

Participants with low perceived stress scores (0–13 on the PSS) were excluded due to their minimal stress levels, which might reduce or eliminate any observable effect of probiotics (33). Existing research indicate that various factors can affect both gut microbiota and stress levels (31); therefore, individuals with chronic illnesses or psychiatric disorders were excluded (34). Pregnant and breastfeeding women were also excluded, as their stress levels, gut microbiota, and dietary habits may differ significantly (35). In addition, smokers (35), as well as individuals who had used medications or supplements within the past 3 months, were not included (36) (Supplementary Figure S1).

### 2.3 Randomization

Eligible participants signed a consent form for their agreement to participate and were informed that their participation would be anonymous. After that, participants were randomized by a simple random sampling method to allocate them into two groups. Each participant was assigned a number, and these numbers were written on identical slips of paper. The papers were thoroughly mixed and randomly drawn to assign participants either to the control group (CG: not receiving anything) or to the group receiving probiotics (PG). All participants attended two visits: Visit 1 (Pre-intervention): Baseline data were collected, including demographic questions, stress levels (PSS), anthropometric measurements and bowel function. Visit 2 (Post-intervention): Stress levels and bowel function were reassessed, and participants were offered incentives in the form of discount vouchers for restaurants and cafes, which they could redeem either during their first or second visit. All questionnaires were administered in both English and Arabic.

### 2.4 Questionnaires

#### 2.4.1 Perceived stress scale

Stress was assessed using the PSS which is a 10-item self-report questionnaire that has been designed to help measure individual stress levels. The tool was developed by Sheldon Cohen and coauthors (32). The questions in the scale ask about feelings and thoughts during the last month (Supplementary material). Five answer options are available for the PSS: 0-never, 1-almost never, 2-sometimes, 3-fairly often, 4-very often. Scoring of the PSS was by first reversing the score for questions 4, 5, 7, and 8 to the following: 0 = 4,1 = 3,2 = 2,3 = 1,4 = 0. Then scores for each item were added up to get the total. PSS scores ranged from 0 to 40 with higher scores indicating higher perceived stress. Scores ranging from 0 to 13 would be considered low stress and were excluded from visit 1. Scores ranging from 14 to 26 would be considered moderate stress, and scores ranging between 27 and 40 would be high perceived stress. The validated English and Arabic versions of the PSS (37) was used and the PSS was collected twice: (pre and post intervention) for both the PG and the CG.

#### 2.4.2 Bowel movements

Bowel function was assessed using a previously validated 21-item questionnaire developed by Zubaidi et al. (38), which was designed according to the Rome criteria and validated in Saudi adults, demonstrating good reliability and internal consistency (Cronbach’s *α* = 0.77).

The questionnaire consisted of 21 questions collecting socio-demographic characteristics, co-morbid illness, medications, and specific questions regarding bowel habits, including fluid intake, usual diet, fecal matter consistency, frequency of defecation, and straining. Other questions in the questionnaire included use of laxatives, previous surgeries, and lifestyle (Supplementary material). For statistical purposes and clear result presentation, some questions were recategorized.

### 2.5 Study intervention

Eligible participants were randomly assigned to either the PG or the CG. The PG consumed one capsule of [*Lactobacillus rhamnosus GG* (LGG), 6 × 10^9^ CFU, Dicoflor 60, manufactured by S.I.I.T. srl, Trezzano sul Naviglio, Milan, Italy, and distributed in Saudi Arabia by Gulf Neo Care, Riyadh].

The probiotic strain is well-documented for its natural resistance to gastric acid and bile, ensuring survival through gastrointestinal transit. The product is a freeze-dried, non–enteric-coated formulation with proven stability when stored below 25 °C as recommended by the manufacturer. Participants consumed the probiotic once daily with food for 30 days and the CG received no intervention. Participants were reminded on a weekly basis to consume their probiotics and were provided with a tracking diary. Participants were instructed to keep forgotten capsules and bring it with them in the second visit. The selection of the probiotic strain, dosage, and duration was based on previous studies (29), the availability of authorized probiotic products at Al-Nahdi® and Amwaj® pharmacies in Saudi Arabia, and cost considerations. The intervention period was limited to 30 days.

### 2.6 Anthropometric measurements and body composition

Body weight and body composition were assessed using a bioelectrical impedance analysis device (Omron HBF-514C, Omron Healthcare Co., Kyoto, Japan). The device provides body composition indices as “levels,” which are categorical interpretations of health-related ranges. For example: body fat percentage is classified into *low, normal, high,* or *very high* ranges based on sex- and age-specific reference values. Visceral fat levels are expressed on a 1–30 scale, where a score of 1–9 indicates normal visceral fat, 10–14 indicates high, and 15–30 indicates very high visceral fat accumulation, which has been associated with increased cardiometabolic risk. Height was measured to the nearest 0.1 cm using a stadiometer. All procedures were conducted according to the WHO protocols for anthropometric measurements (39) to ensure standardization and reproducibility.

### 2.7 Statistical analyses

#### 2.7.1 Sample size calculation

Sample size estimation was conducted using G*Power (version 3.1.9.6) for a multiple linear regression model to detect a moderate effect size (*f^2^* = 0.15) with an α-level of 0.05 and power of 0.80. The analysis was designed to test the incremental contribution of one predictor (e.g., intervention group) after accounting for four covariates (i.e., a total of five predictors in the model). The result indicated that a total sample size of 55 participants would be required to detect a statistically significant change in the outcome variable. To further ensure adequate statistical power, we oversampled by approximately 15–20% to account for attrition, incomplete data, or non-adherence to the intervention protocol. This strategy was intended to preserve the minimum required sample size for the primary analysis and maintain robustness of the regression model.

#### 2.7.2 Statistical tests

Descriptive statistics (mean ± standard deviation for continuous variables; frequencies and percentages for categorical variables) were used to summarize participant characteristics at baseline. Group differences in categorical variables, such as sex and BMI category, were examined using the chi-square test of independence. Mean difference in stress scores from baseline to endline were assessed using Wilcoxon rank-sum. Independent two-sample t-test was applied to compare the mean of continuous variables, including endline stress scores, between the intervention and control groups. Sex-stratified descriptive comparisons were pre-planned and conducted for exploratory purposes. However, the study was not powered to detect sex × treatment interaction effects. To further account for baseline stress scores (40) and potential confounders (age, sex, BMI (29)), a multiple linear regression model was fitted with endline stress score as the dependent variable. Regression coefficients (β) and corresponding *p*-values were reported, and statistical significance was defined as *p* < 0.05. Logistic regression analyses were conducted to examine associations between bowel movement characteristics and group status (probiotic vs. control) at both baseline and post-intervention. All statistical analyses were conducted using Stata 14.2 SE for Mac.

## 3 Results

**Figure 1:**
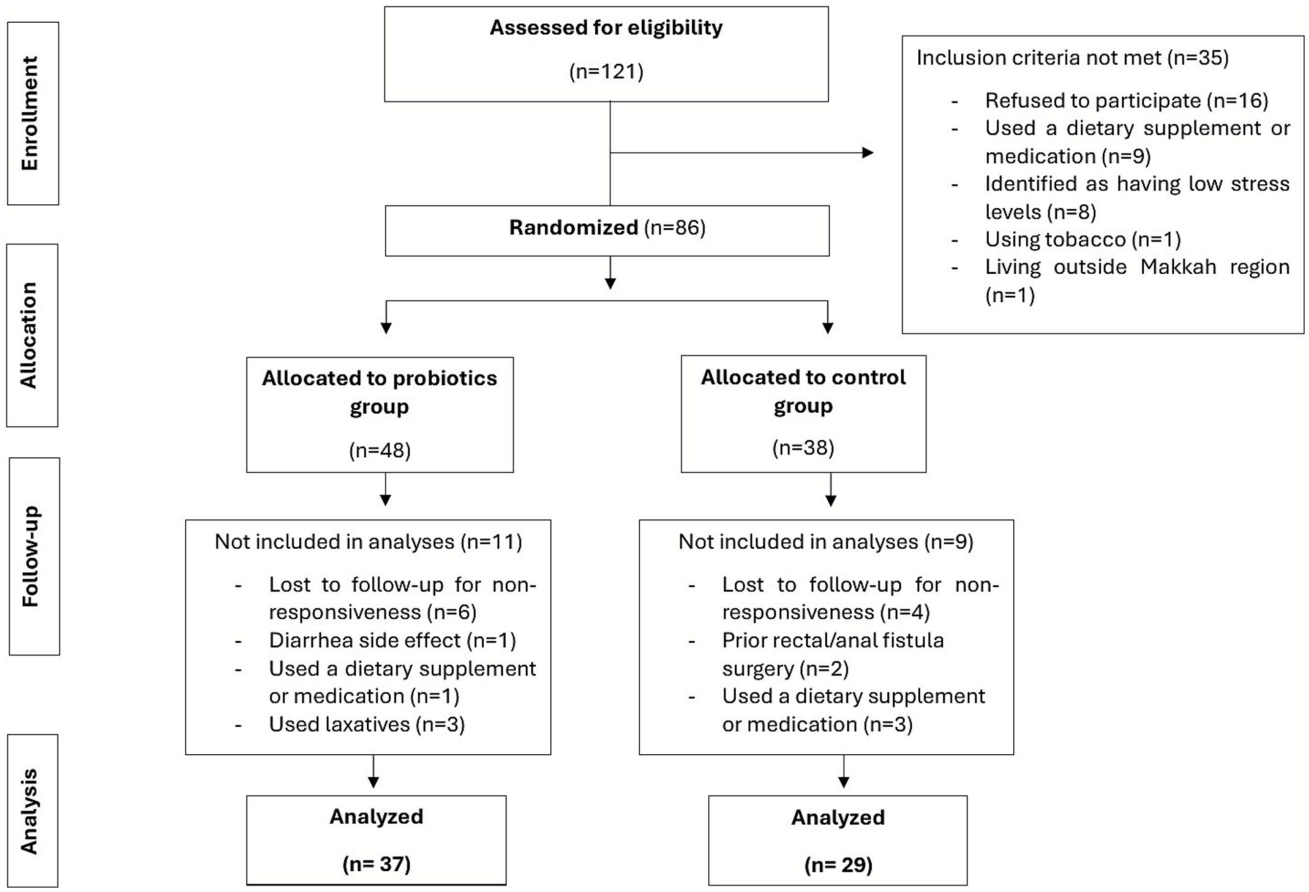
CONSORT flow diagram of participant enrollment, allocation, follow-up, and analysis in the trial.

Figure 1 illustrates the flow of participants from eligibility screening through randomization, allocation to intervention or control, follow-up, and final analysis, including reasons for exclusion at each stage. Participant adherence to the probiotic regimen was excellent throughout the study. All participants were reminded weekly via WhatsApp messages to ensure capsule consumption, and all bottles were returned empty at the end of the 30-day intervention period. As such, no missed doses were reported, and all participants were fully compliant with the supplementation protocol. No participants were excluded due to poor adherence; however, as shown in Figure 1, one participant was excluded due to side effects, and others were excluded because of the use of medications, supplements, laxatives, or recent surgeries. Additional participants were lost to follow-up due to non-responsiveness when scheduling the post-intervention assessment.

### 3.1 Baseline characteristics

At baseline, the probiotic group (n *=* 37) had a mean age of 21.97 ± 2.59 years, while the control group (n *=* 29) had a mean age of 20.83 ± 1.91 years (*p* = 0.05). In terms of sex distribution, the probiotic group comprised 56.8% males and 43.2% females, whereas the control group included 51.7% males and 48.3% females (*p* = 0.493). No significant differences were observed between groups in body fat percentage, visceral fat, stress scores, BMI, stress level categories, physical activity, education level, or occupation (all *p* > 0.05). The only significant difference was in type of residence, with more participants in the control group residing in apartments compared to the probiotic group (*p* = 0.044) (Table 1).

**Table 1:** Baseline characteristics of the study groups.

Variables	Probiotic group (*n* = 37)	Control group (*n* = 29)	*p* value
Sex			0.493
Males	21 (56.8%)	15 (51.7%)	
Females	16 (43.2%)	14 (48.3%)	
Age, years (mean ± SD)	21.97 ± 2.59	20.83 ± 1.91	0.050
Body Fat % (mean ± SD)	30.08 ± 12.40	31.89 ± 11.23	0.541
Visceral fat (level)	6.54 ± 4.59	6.03 ± 4.49	0.655
Total stress score	23.16 ± 4.56	23.86 ± 4.03	0.518
Stress level			0.858
Low	-	-	
Moderate	30 (81.1%)	23 (79.31%)	
High	7 (18.9%)	6 (20.69)	
BMI (kg/m^2^)	25.42 ± 6.03	24.84 ± 6.00	0.699
BMI category, *n* (%)			0.505
Underweight	2 (5.4%)	5 (17.2%)	
Healthy weight	18 (48.6%)	14 (48.3%)	
Overweight/Obese	17 (45.9%)	10 (34.5%)	
Physical activity, *n* (%)			0.628
Daily/few times per week	12 (32.4%)	11 (37.9%)	
Weekly/few times per month	13 (35.1%)	7 (24.1%)	
No specific time	12 (32.4%)	11 (37.9%)	
Education level, *n* (%)			0.493
High school	7 (18.9%)	3 (10.3%)	
University	29 (78.4%)	26 (89.7%)	
Postgraduate	1 (2.7%)	0 (0.0%)	
Occupation, *n* (%)			0.279
Student	30 (81.1%)	27 (93.1%)	
Not student	7 (18.9%)	2 (6.9%)	
Type of residence, *n* (%)			0.044
Apartment/Flat	15 (40.54%)	19 (65.5%)	
House	22 (59.5%)	10 (34.5%)	

Data are presented as mean ± SD and *n* (%).

### 3.2 Total stress scores

**Figure 2:**
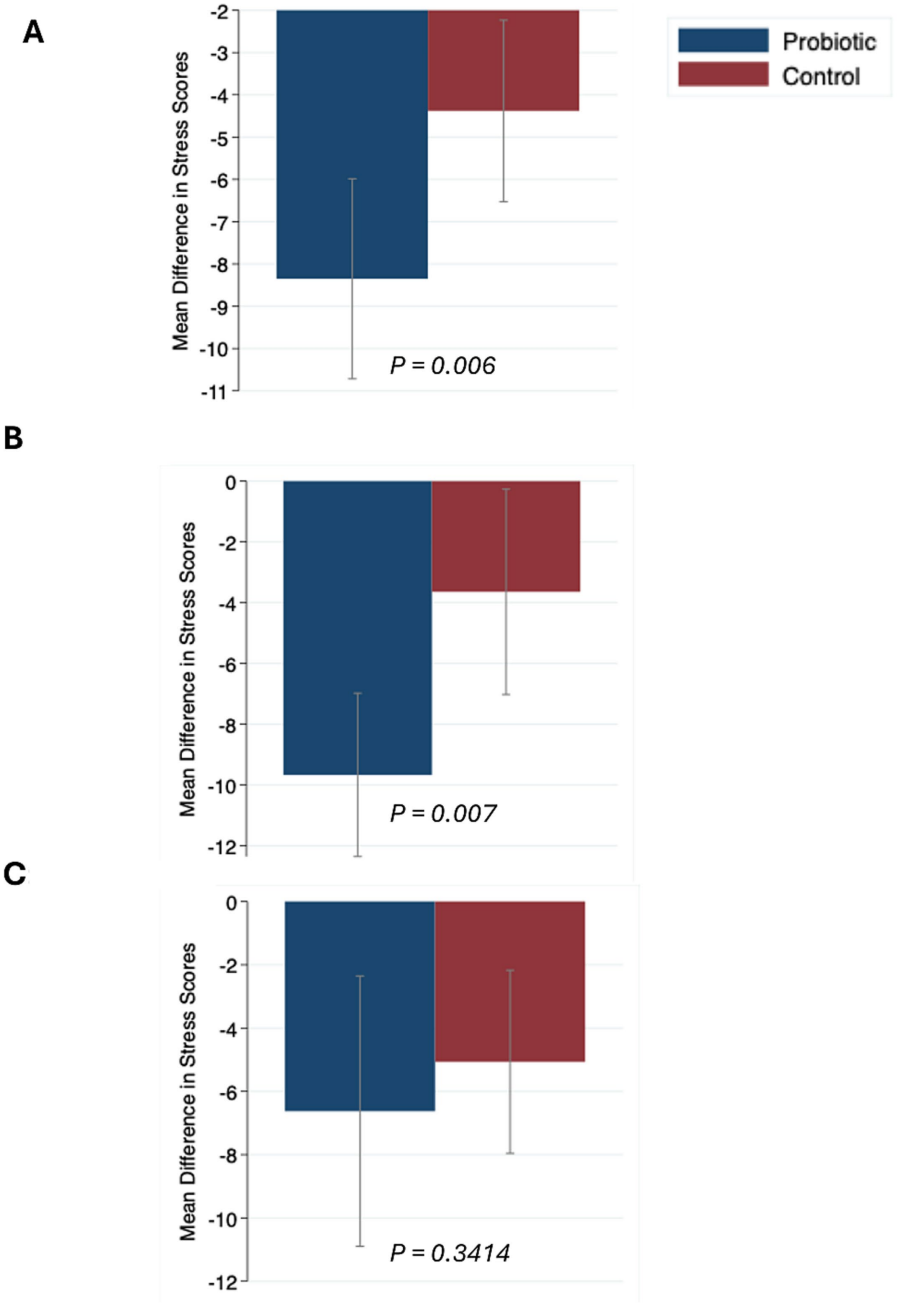
Mean (95%CI) changes pre- and post-intervention in the probiotic and control groups for **(A)** total sample (*n* = 66), **(B)** Males, **(C)** Females. Data were compared using Wilcoxon rank-sum.

Mean change in the probiotic and control group is shown in Figure 2. Probiotics significantly reduced stress in the total sample (Figure 2A) and in males Figure 2B (See Figure 2), but showed no significant effect in females (Figure 2C, *p* = 0.3414). A significant reduction in mean stress scores from baseline to post-intervention in both the probiotic group (*p* < 0.001) and the control group (*p* < 0.001) was observed (data not shown). There were not significant sex-differences at baseline stress scores in the probiotic group (23.8 ± 4.6 vs. 22.7 ± 4.6) or the control group (23.8 ± 4.2 vs.23.9 ± 4.0).Table 2 shows the stress scores post-intervention between the PG and CG and by sex. In the total sample, post-intervention stress scores were significantly lower in the probiotic group compared with the control group (14.81 ± 6.12 vs. 19.48 ± 5.91; *p* = 0.003). Within-group sex comparisons showed that males in PG had lower stress scores than females (13.00±4.92 vs. 17.19± 6.86, *p* = 0.037), whereas in CG, males did not differ than females (20.29±56.46 vs. 18.73±5.46, *p* = 0.49). Regarding stress level categories (low, moderate, high), the distribution did not differ significantly between sexes in either group (all *p* > 0.05). However, in the total sample, there was a significant difference between the probiotic and control groups (*p* = 0.008), with a higher proportion of participants in the probiotic group classified as having low stress (84.2% vs. 15.8% in controls).

**Table 2:** Post-intervention comparison of stress scores and stress categories between PG and CG stratified by sex.

Variable	Control group (CG)			*p* value (sex-comparisons)	Probiotic group (PG)			*p* value (sex-comparisons)	PG vs. CG *p* value (total)
	Total	females	males		Total	females	males		
	*n* = 29	*n* = 15	*n* = 14		*n* = 37	*n* = 16	*n* = 21		
Stress scores	19.48 ± 5.91	18.73 ± 5.46	20.29 ± 6.46	0.490	14.81 ± 6.12	17.19 ± 6.86	13.00 ± 4.92	0.037	0.003
Stress level									
Low	3 (15.8%)	1 (33.3%)	2 (66.7%)	0.792	16 (84.2%)	5 (31.3%)	11 (68.7%)	0.188	0.008
Moderate	24 (55.8%)	13 (54.2%)	11 (45.83%)		19 (44.2%)	9 (47.4%)	10 (52.6%)		
High	2 (50%)	1 (50%)	1 (50%)		2 (50%)	2 (100%)	0 (0.0%)		

Data is presented as mean ± SD or *n* (%). Two sample t-test was used for continuous data and chi-squared test for categorical variables.

### 3.3 Association between probiotic supplementation and post-intervention stress scores

Data (*n* = 66) were analyzed using multiple linear regression, adjusting for group allocation, baseline stress scores, age, sex, and baseline BMI (Table 3). Participants in the control group had mean stress scores that were 3.79 points higher than those in the probiotic group (β = 3.79, 95% CI: 0.74, 6.83; *p* = 0.016). These results suggest that probiotic supplementation independently predicted lower stress scores after the intervention, regardless of participants’ age, sex, BMI and baseline stress scores.

**Table 3:** Multiple linear regression analysis of post-intervention stress scores and associated variables.

Post-intervention total stress scores	β (95% CI)	*p*-value
Group (control vs. intervention)	3.79 (0.74,6.83)	0.016
Baseline Stress Scores	0.3 (−0.05,0.64)	0.09
Age, y	−0.48 (−1.13,0.17)	0.141
Sex (male vs. female)	−1.44 (−4.42,1.54)	0.338
Baseline BMI, kg/m^2^	0 (−0.25,0.25)	0.996

Data (*n* = 66) were analyzed using multiple linear regression, adjusting for group allocation, baseline stress scores, age, sex, and baseline BMI. 21.6% of the variance in post-intervention total stress scores (R^2^ = 0.216; adjusted R^2^ *=* 0.151).

### 3.4 Bowel movement at baseline and post-intervention

At baseline, both groups were largely similar in bowel movement characteristics (Table 4). Most participants reported having a normal bowel movement (probiotic: 75.7%, control: 89.7%; *p* = 0.203) and defecated once daily (probiotic: 59.5%, control: 62.1%; *p* = 0.736). Around half experienced problems after eating certain foods (probiotic: 51.4%, control: 44.8%; *p* = 0.599), most commonly gas, diarrhea, or pain. Control problems were rare (13.5% in both groups; *p* = 0.974). Daily liquid intake was the only measure approaching significance (*p* = 0.062), with 48.7% of the probiotic group drinking only 1–4 cups per day compared to 27.6% in the control group. Overall, no statistically significant baseline differences were found.

**Table 4:** Bowel movement at baseline for PG and CG.

Variables	Probiotic group (*n* = 37)	Control group (*n* = 29)	*p* value
Do you think you have a normal bowel movement?			0.203
Yes	28 (75.68%)	26 (89.66%)	
No	9 (24.32%)	3 (10.34%)	
The amount of daily liquids			0.062
1–4 cups	18 (48.65%)	8 (27.59%)	
4–6 cups	7 (18.92%)	13 (44.83%)	
>6 cups	12 (32.43%)	8 (27.59%)	
Frequency of defecation			0.736
Once/day	22 (59.46%)	18 (62.07%)	
Once/week	1 (2.70%)	2 (6.90%)	
More than once a week	4 (10.81%)	4 (13.79%)	
3–7 times/week	10 (27.03%)	5 (17.24%)	
I have problems after eating certain foods			0.599
No	18 (48.65%)	16 (55.17%)	
Yes	19 (51.35%)	13 (44.83%)	
These problems are			0.606
No issues	17 (45.95%)	14 (48.28%)	
Gas/Diarrhea/Pain	10 (27.03%)	10 (34.48%)	
More than one answer	10 (27.03%)	5 (17.24%)	
I have a control problem			0.974
No	32 (86.49%)	25 (86.21%)	
Yes	5 (13.51%)	4 (13.79%)	
Difficulties during defecation			0.346
No	27 (72.97%)	24 (82.76%)	
Yes	10 (27.03%)	5 (17.24%)	

Data is presented in numbers of participants and percentages; Chi Square or Fisher’s Exact tests were used.

Post-intervention, bowel movement characteristics were generally similar between the probiotic and control groups (Table 5). Most participants in both groups perceived their bowel movements as normal (probiotic: 91.9%, control: 89.7%; *p* = 1.000) and had comparable defecation frequencies (*p* = 0.936). Problems after eating certain foods were reported by 35.1% of the probiotic group and 48.3% of the control group (*p* = 0.281), with 70.3% of probiotic participants versus 48.3% of controls reporting no issues (*p* = 0.178). Control problems remained rare (8.1% vs. 6.9%; p = 1.000). Daily liquid intake was the only measure approaching statistical significance (*p* = 0.051), with 45.9% of probiotic participants consuming 1–4 cups/day compared to 24.1% of controls. Overall, no statistically significant differences were observed. Logistic regression showed that baseline and post-intervention bowel movement characteristics were similar between the probiotic and control groups. Participants reporting a daily liquid intake of 4–6 cups had significantly lower odds of reporting probiotic use compared with those consuming 1–4 cups *p* < 0.05 (Supplementary Tables S1, S2 respectively). No other baseline variables showed significant associations with group status.

**Table 5:** Bowel movement at post-intervention for PG and CG.

Variables	Probiotic group (*n* = 37)	Control group (*n* = 29)	*p* value
Do you think you have normal bowel movement?			1.000
Yes	34 (91.89%)	26 (89.66%)	
No	3 (8.11%)	3 (10.34%)	
The amount of daily liquids			0.051
1–4 cups	17 (45.95%)	7 (24.14%)	
4–6 cups	6 (16.22%)	12 (41.38%)	
>6 cups	14 (37.84%)	10 (34.48%)	
Frequency of defecation			0.936
Once/day	13 (35.14%)	9 (31.03%)	
Once/week	4 (10.81%)	2 (6.90%)	
More than once a week	9 (24.32%)	9 (31.03%)	
3–7 times/week	11 (29.73%)	9 (31.03%)	
I have problems after eating certain foods			0.281
No	24 (64.86%)	15 (51.72%)	
Yes	13 (35.14%)	14 (48.28%)	
These problems are			0.178
No issues	26 (70.27%)	14 (48.28%)	
Gas/Diarrhea/Pain	6 (16.22%)	7 (24.14%)	
More than one answer	5 (13.51%)	8 (27.59%)	
I have a control problem			1.000
No	34 (91.89%)	27 (93.10%)	
Yes	3 (8.11%)	2 (6.90%)	
Difficulties during defecation			0.384
No	18 (48.65%)	11 (37.93%)	
Yes	19 (51.35%)	18 (62.07%)	

Data is presented in numbers of participants and percentages; Chi Square or Fisher’s exact tests were used.

## 4 Discussion

To our knowledge, this is the first RCT studying the effects of probiotic on stress and bowel movements in stressed healthy Saudi young adults. PSS decreased after 30 days from baseline in both probiotic and control groups for both males and females. Post-intervention, stress scores were significantly lower in the probiotic group compared with the control group with the effect being significant in males but not females. In the total sample, post-intervention, a greater proportion of the probiotic group had low stress levels compared to controls. In adjusted analyses, participants in the control group had significantly higher post-intervention stress scores than those in the probiotic group (by 3.79 points; *p* = 0.016). No other variables were significantly associated with stress scores, indicating that probiotic supplementation independently predicted lower stress. At baseline, bowel movement patterns were similar between groups, with daily liquid intake nearly reaching significance. Post-intervention, no significant differences emerged, though the probiotic group tended to report fewer food-related problems.

Both the probiotic and control groups exhibited decreased stress levels post-intervention consistent with other studies (28, 41, 42) and that may be attributed to non-treatment-specific factors. First, the placebo effect can meaningfully reduce subjective stress through positive expectations alone (43). Although our study included a control group, participants did not receive a placebo treatment. This design was chosen to compare the probiotic intervention directly against natural progression without introducing additional external influences. However, the absence of a placebo means that non-specific psychological effects, such as expectancy or perceived care, cannot be fully ruled out as contributors to the observed reduction in stress scores in both groups. The post-intervention decreases in stress scores observed in both groups may reflect natural changes over time rather than treatment effects. Since most participants were university students assessed during the same academic cycle, reductions may be linked to the passing of peak academic stressors such as examinations or assignment deadlines, as reported in student populations (44). Participants were recruited over two academic years, during which UQU followed a three-semester system, allowing recruitment across different academic contexts. To minimize potential timing bias, all participants were recruited and completed the probiotic intake phase before Ramadan, as this period involves substantial changes in eating and sleeping patterns that could affect both stress and gut microbiota composition. Post-intervention assessments varied slightly depending on recruitment timing but were predominantly conducted after Ramadan, typically following the final examination period and the Eid holiday—an interval characterized by reduced academic and psychological stress. Baseline stress levels were included as covariates in the multiple regression analysis to further account for timing effects and natural fluctuations in stress across the academic cycle. Together, these factors may explain the general decline in stress across both groups and strengthen confidence that the observed outcomes reflect true intervention effects beyond timing-related variation. Moreover, psychological adaptation, resilience, and study participation effects—such as increased self-awareness or subtle lifestyle modifications—may also have contributed to reductions in perceived stress despite the absence of a placebo condition (45). Although researchers explicitly advised participants not to change their daily routines during the trial, it is possible that the mere act of study participation encouraged some modifications—such as improved sleep hygiene or diet—that indirectly reduced stress in the control group.

In this trial, probiotic supplementation led to significantly greater reductions in perceived stress than placebo, including a 3.79 point adjusted difference (95% CI 0.74–6.83; *p* = 0.016). Bar plots (Figure 2) confirmed larger reductions overall (*p* = 0.006) and in males (*p* = 0.007), with no significant effect in females (*p* = 0.3414). These results suggest probiotics may provide a modest but meaningful stress-reducing benefit in healthy young adults. These results align with growing evidence from human trials and meta-analyses indicating that certain probiotic strains can alleviate subjective stress and mood symptoms (27, 29, 46–50). A meta-analysis of seven RCTs in healthy, stressed adults found probiotics reduced subjective stress (29). However, subgroup analyses by product type or duration were null, likely due to heterogeneity in stress measures which differ in sensitivity and construct coverage; psychometric work shows overlap with fatigue/depressiveness and limited equivalence across tools (51). We used the PSS because it is the only Arabic-validated instrument, ensuring cultural/linguistic validity. Differences in sample size/power across studies further dilute pooled effects. Null subgroups may also reflect inter-individual microbiota variability and the lack of functional readouts in trials (52). Finally, effects are strain-specific; some Lactobacillus/Bifidobacterium combinations improve depressive symptoms on certain scales but not others indicating that both strain composition and outcome measure shape observed results (53).

Our trial showed a reduction in perceived stress versus controls after 30 days in males. By contrast, Guan et al. (42) tested a fermented-milk product containing *Lacticaseibacillus paracasei* K56 for 2 weeks in master’s/doctoral students and found no between-group effect on PSS-10, although secondary outcomes improved (lower stress/anxiety, better sleep), with higher serum 5-hydroxytryptamine (serotonin), greater *Lacticaseibacillus* abundance, and increased butyrate—suggesting biological change without a detectable PSS signal over that short window. In a different population, Sanchez et al. (54) reported sex-dependent responses to *Lactobacillus rhamnosus* during a 24-week weight-reduction program: women showed clearer mood/behavioral benefits, while some appetite-related effects occurred in men, underscoring that probiotic efficacy can vary by sex. Mechanistically, a recent review highlights multiple sex-linked pathways along the microbiota–gut–brain axis (55). First, baseline microbiome differences between males and females (including differing prevalence of *Lactobacillus*/*Bifidobacterium*), second, cross-talk with sex hormones that shapes immune tone and HPA-axis reactivity, third, neural signaling (e.g., vagal pathways), and metabolites (e.g., Short Chain Fatty Acids (SCFAs)) that influence stress physiology. These “microgenderome” features offer plausible bases for sex-dependent benefits in our cohort and for null PSS findings in Guan et al. (42) despite biological changes, especially given differences in product matrix/delivery (capsules vs. fermented milk) and exposure time (30 days vs. 14 days), which can alter the magnitude and timing of perceptible effects on subjective stress.

In contrast to the present trial, several human studies have reported different effects on PSS scores (42, 56, 57). For example, Guan et al. observed a significant reduction in PSS in the probiotic arm versus placebo at week 1; by week 2, both groups had improved and the between-group difference was no longer significant. Methodological and cohort heterogeneity likely account for these discrepancies. First, sample size differed (*n* = 66 in our study vs. *n* = 120 in theirs), which affects precision and detectable effect sizes. Second, our sample comprised predominantly undergraduate students, whereas their sample included only master’s and doctoral students—populations that differ in academic demands, schedules, and leave patterns—and the mean age also differed (20–21 years in our study vs. 24 years in theirs; Table 1), factors that can influence stress trajectories and intervention responsiveness. Third, intervention characteristics varied: we administered probiotics for 30 days without a placebo control, while their trial delivered a dairy-based product for 2 weeks with a placebo comparator. Finally, the probiotic strains were not the same—our study used *Lactobacillus rhamnosus*, whereas theirs used *Lacticaseibacillus paracasei* K56—and probiotic effects are known to be strain-specific, with different strains demonstrating distinct physiological actions and clinical outcomes (53).

Findings from Messaoudi et al. (56) differ from ours: in their 30-day RCT of *Lactobacillus helveticus* R0052 + *Bifidobacterium longum* R0175 (“Probio’Stick”) no between-group differences in PSS were detected at baseline or over time. Their product was delivered as a 1.5-g stick/sachet (3 × 10^9 CFU/day) taken during or just after breakfast for 30 days, with 55 participants completing the trial and only 7 men per arm. By contrast, our study (*n* = 66) showed significant change from baseline and between-group differences in males, which is plausible because the comparator trial was under-powered to detect male-specific effects (7/arm) and sex differences in stress-system reactivity are well documented (58). Moreover, Messaoudi et al. noted that PSS may be less sensitive over a 30-day window compared with other scales, which could mask treatment effects. Formulation and delivery route also likely contributed. Their probiotics were given as an uncoated powder stick with food, whereas we used capsules. Oral delivery format influences the number of viable cells that reach the small intestine (59, 60):delayed release/enteric systems and microencapsulation (61) consistently improve survival through gastric acid versus powders or standard capsules, and they can enhance downstream functional readouts. In addition, administration timing and food matrix alter survival; giving probiotics with a meal or 30 min before (particularly meals with some fat or a protective matrix like oatmeal-milk) improves survivability compared with water/juice or dosing after a meal (62). The co-ingested matrix independently modulates viability during gastric transit (63). Finally, the strains differed between studies (our capsule contained a different strain) and probiotic effects are strain-specific; clinical benefits with one strain cannot be assumed for another (53). Notably, exclusion criteria were broadly comparable (e.g., exclusion of neuropsychiatric/major medical conditions, psychotropic drugs, pregnancy), supporting that cohort differences are unlikely to fully account for divergent outcomes. Taken together—sex-specific power, outcome sensitivity, and differences in probiotic delivery (dosage form, timing, and matrix) and strain identity—offer coherent explanations for why Messaoudi et al. observed no PSS effect while we detected significant improvements in men.

We limited enrollment to participants with moderate–high perceived stress to maximize sensitivity and avoid floor/ceiling effects. Psychobiotic RCTs that enrich for elevated baseline stress have shown clearer signals—for example, a double-blind trial in nurses required PSS ≥ 27 at screening and, while many outcomes improved in both arms, the probiotic produced a between-group reduction in cortisol and benefits in the high-anxiety subgroup, indicating effects are easier to detect when baseline distress is higher (33). In student populations, recent trials recruited “moderate stress” samples and even stratified randomization by baseline PSS, reflecting the same design logic and the standard PSS bands (0–13 low, 14–26 moderate, 27–40 high) (42). Finally, reviews emphasize that heterogeneous samples and short interventions can dilute observed effects, reinforcing the value of stress-enriched cohorts for detecting psychobiotic benefits (64).

Our adjusted models indicate that probiotics lowered perceived stress, but residual confounding is still possible. Unmeasured factors such as menstrual-cycle phase (65), habitual diet (especially fiber/fermented foods that shift microbiota and SCFA output) (66), sleep/physical activity (67) or caffeine use, and host genetics (68) could all influence stress biology and probiotic response. Mechanistically, probiotics can act along the microbiota–gut–brain axis via neural (vagus), endocrine and immune (cytokine) routes, and by altering tryptophan–kynurenine metabolism and SCFA signaling—each linked to stress reactivity (23). Probiotics also show antioxidant actions in humans (69) and turn on the cell’s built-in antioxidant system (70), offering a plausible route to dampen stress physiology. Together, these mechanisms—and the unmeasured behavioral/biological moderators above—help explain similarities and differences across studies and may also contribute to subgroup effects in our trial.

Our trial showed no between-group change in bowel habits, a result that sits within mixed findings from studies measuring stress and GI outcomes together. In medical students, fermented milk with *Lactobacillus casei Shirota* reduced abdominal dysfunction and blunted exam-stress cortisol, though GI effects were modest overall (71). Paraprobiotic *Lactobacillus gasseri* CP2305 improved stress symptoms and stool properties in student cohorts (72). In pre-graduate students, *Lacticaseibacillus paracasei* K56 produced within-group Gastrointestinal Symptom Rating Scale (GSRS) improvements with rises in serum 5-HT and fecal butyrate, but limited PSS change—suggesting mechanistic shifts can precede noticeable bowel-habit change (42). In IBS, *Bifidobacterium longum* NCC3001 improved depressive symptoms and provided adequate relief of IBS symptoms, underscoring gut–brain links in patients (73). Differences from our null GI result likely reflect: (1) near-normal baseline bowel function (2) 30-day exposure (shorter than some positive trials), (3) strain differences (our capsules vs. several fermented-milk products), (4) sample size not powered for small motility changes, (5) use of a validated Saudi bowel-habit tool that may be less sensitive than GSRS for short-term shifts, and (6) unmeasured diet, sleep/activity, hydration, and menstrual-cycle phase—all of which can modulate the microbiome and stress biology (66).

This is, to our knowledge, the first trial in Saudi adults examining probiotic effects on perceived stress, and it used multivariable regression (beyond simple group comparisons) to adjust for key covariates. Additional strengths include a pre-specified power calculation, use of a validated Arabic PSS, sex-stratified analyses and adherence reminders. This study has some limitations that should be acknowledged. First, the absence of a placebo group limited our ability to implement blinding procedures. Despite multiple attempts to obtain or produce suitable placebo capsules through local pharmaceutical companies and academic institutions, none were able to manufacture capsules identical in appearance and formulation to the probiotic supplement. Consequently, participant and investigator blinding were not feasible. The lack of placebo and blinding may have introduced some degree of expectation bias; however, these constraints were unavoidable given the logistical and technical challenges of conducting the first randomized controlled probiotic trial among young adults in Saudi Arabia. Future research should aim to include placebo-controlled, double-blind designs to enhance methodological rigor and strengthen the validity of the findings. The absence of a placebo and blinding may limit the ability to fully distinguish the probiotic effect from expectancy or participation influences. To minimize bias, we performed multiple regression analyses adjusting for baseline stress, age, sex, and BMI, and applied strict exclusion criteria to control for factors affecting stress and gut microbiota (e.g., pregnancy, smoking, medication use, and chronic illness). Although these measures strengthened internal validity, they made participant recruitment challenging and resulted in a smaller sample size. Although sex-stratified analyses were performed, the study was not powered or designed to detect interaction effects. Future placebo-controlled, double-blind studies with larger sample sizes exploring sex × treatment are recommended to confirm these findings. Further limitations are the short intervention period (30 days), no assessment of dietary intake (e.g., fiber/fermented foods) or biomarkers (e.g., cortisol, SCFAs, 5-HT), and reliance on self-report GI measures—factors that may have reduced sensitivity to detect bowel-habit changes and limited mechanistic inference.

Our findings suggest this probiotic can be considered as an adjunct for reducing stress in especially those with moderate to high baseline stress, while expectations for short-term changes in bowel habits should remain modest. In practice, use validated Arabic measures for follow-up, emphasize adherence, and couple the probiotic with guidance on sleep, physical activity, and a diet rich in fiber and fermented foods. Future studies should be larger and longer, placebo-controlled and double-blind, and powered to detect sex-by-treatment effects. They should track dietary intake, sleep, menstrual cycle, medications, and hydration; include mechanistic biomarkers such as cortisol, SCFAs, and serotonin, alongside microbiome composition and functional readouts; use standardized GI scales like the GSRS and the Bristol Stool Form Scale and compare strains, doses, and delivery formats to identify who benefits most.

## 5 Conclusion

Thirty days of probiotic supplementation reduced perceived stress compared with controls, while bowel habits remained unchanged. Probiotics may serve as a potential adjunctive strategy for stress management in males, pending confirmation in larger and longer placebo-controlled trials. These findings underscore the relevance of the gut-brain axis in stress modulation and support further investigation into strain-specific efficacy and mechanistic pathways.

## Data Availability

The data supporting the findings of this study are available from the corresponding author upon reasonable request.

## References

[1] WHO. (2023). Stress. Available online at: https://.www.who.int/news-room/questions-and-answers/item/stress [Accessed June 8, 2025].

[2] National Institute of Mental Health (2020). I’m so stressed out! Fact sheet. NIH publication no 20-MH-8125. Available online a: thttps://www.nimh.nih.gov/health/publications/so-stressed-out-fact-sheet [Accessed June 8, 2025].

[3] Van Der Molen HF Nieuwenhuijsen K Frings-Dresen MHW De Groene G. Work-related psychosocial risk factors for stress-related mental disorders: an updated systematic review and meta-analysis. BMJ Open. (2020) 10:4849. doi: 10.1136/bmjopen-2019-034849, 32624469 PMC7337889

[4] Kim HG Cheon EJ Bai DS Lee YH Koo BH. Stress and heart rate variability: a meta-analysis and review of the literature. Psychiatry Investig. (2018) 15:235–45. doi: 10.30773/pi.2017.08.17, 29486547 PMC5900369

[5] Hill D Conner M Clancy F Moss R Wilding S Bristow M . Stress and eating behaviours in healthy adults: a systematic review and meta-analysis. Health Psychol Rev. (2022) 16:280–304. doi: 10.1080/17437199.2021.1923406, 33913377

[6] World Health organization. (2023). Stress. Available online at: https://applications.emro.who.int/docs/WHOEMMNH236E-eng.pdf [Accessed June 8, 2025].

[7] Stubbs B Veronese N Vancampfort D Prina AM Lin P-Y Tseng P-T . Perceived stress and smoking across 41 countries: a global perspective across Europe, Africa, Asia and the Americas. Sci Rep. (2017) 7:7597. doi: 10.1038/s41598-017-07579-w, 28790418 PMC5548752

[8] Blaine SK Sinha R. Alcohol, stress, and glucocorticoids: from risk to dependence and relapse in alcohol use disorders. Neuropharmacology. (2017) 122:136–47. doi: 10.1016/j.neuropharm.2017.01.037, 28159647 PMC5479733

[9] Santos J Maran PL Rodríguez-Urrutia A. Stress, microbiota, and the gut–brain axis in mental and digestive health. Med Clin (Barc). (2025) 164:295–304. doi: 10.1016/j.medcli.2024.11.023, 39824687

[10] Richardson S Shaffer JA Falzon L Krupka D Davidson KW Edmondson D. Meta-analysis of perceived stress and its association with incident coronary heart disease. Am J Cardiol. (2012) 110:1711–6. doi: 10.1016/j.amjcard.2012.08.004, 22975465 PMC3511594

[11] Liu M-Y Li N Li WA Khan H. Association between psychosocial stress and hypertension: a systematic review and meta-analysis. Neurol Res. (2017) 39:573–80. doi: 10.1080/01616412.2017.1317904, 28415916

[12] Tomiyama AJ. Stress and obesity. Annu Rev Psychol. (2019) 70:703–18. doi: 10.1146/annurev-psych-010418-102936, 29927688

[13] Smith MD Wesselbaum D. Global evidence on the prevalence of and risk factors associated with stress. J Affect Disord. (2025) 374:179–83. doi: 10.1016/j.jad.2025.01.053, 39805499

[14] WHO. (2022). Mental health at work: policy brief. Available online at: https://www.who.int/publications/i/item/9789240057944 [Accessed June 12, 2025].

[15] Zuberi A Waqas A Naveed S Hossain MM Rahman A Saeed K . Prevalence of mental disorders in the WHO eastern Mediterranean region: a systematic review and meta-analysis. Front Psychol. (2021) 12:665019. doi: 10.3389/fpsyt.2021.665019, 34335323 PMC8316754

[16] Jeyapalan T Blair E. The factors causing stress in medical students and their impact on academic outcomes: a narrative qualitative systematic review. Int J Med Students. (2024) 12:195–203. doi: 10.5195/ijms.2024.2218

[17] Alosaimi FD Kazim SN Almufleh AS Aladwani BS Alsubaie AS. Prevalence of stress and its determinants among residents in Saudi Arabia. Saudi Med J. (2015) 36:605–12. doi: 10.15537/smj.2015.5.10814, 25935183 PMC4436759

[18] Almutairi AF Hamdan N Altheyabi S Alsaeed E Alammari F BaniMustafa A. The prevalence and associated factors of occupational stress in healthcare providers in Saudi Arabia. Int J Gen Med. (2024) 17:809–16. doi: 10.2147/ijgm.s446410, 38476624 PMC10929258

[19] Abdoh DS Shahin MA Ali AK Alhejaili SM Kiram OM Al-Dubai SAR. Prevalence and associated factors of stress among primary health care nurses in Saudi Arabia, a multi-center study. J Family Med Prim Care. (2021) 10:2692–6. doi: 10.4103/jfmpc.jfmpc_222_21, 34568156 PMC8415654

[20] Alsaleem MA Alsaleem SA Al Shehri S Awadalla NJ Mirdad TM Abbag FI . Prevalence and correlates of university students’ perceived stress in southwestern Saudi Arabia. Medicine (Baltimore). (2021) 100:7295. doi: 10.1097/MD.0000000000027295, 34559140 PMC8462648

[21] Alamri HS Algarni A Shehata SF Bshabshe A Al Alshehri NN . Prevalence of depression, anxiety, and stress among the general population in Saudi Arabia during covid-19 pandemic. Int J Environ Res Public Health. (2020) 17:1–11. doi: 10.3390/ijerph17249183 PMC7764434 33316900

[22] Dabbagh R Alwatban L Alrubaiaan M Alharbi S Aldahkil S AlMuteb M . Depression, stress, anxiety and burnout among undergraduate and postgraduate medical trainees in Saudi Arabia over two decades: a systematic review. Med Teach. (2023) 45:499–509. doi: 10.1080/0142159X.2022.2139669, 36355388

[23] Cryan JF Dinan TG. Mind-altering microorganisms: the impact of the gut microbiota on brain and behaviour. Nat Rev Neurosci. (2012) 13:701–12. doi: 10.1038/nrn3346, 22968153

[24] Khatun MT Hoque F Hoque NS Hossain MS Alam MA Afrin S . Emerging role of probiotics in advancement of combating physical abnormalities and diseases: a systematic perspective analysis. Asian J Biochem Genet Molecul Biol. (2024) 16:1–23. doi: 10.9734/ajbgmb/2024/v16i8397

[25] Mindus C Ellis J van Staaveren N Harlander-Matauschek A. *Lactobacillus*-based probiotics reduce the adverse effects of stress in rodents: a meta-analysis. Front Behav Neurosci. (2021) 15:642757. doi: 10.3389/fnbeh.2021.642757, 34220459 PMC8241911

[26] Chao L Liu C Sutthawongwadee S Li Y Lv W Chen W . Effects of probiotics on depressive or anxiety variables in healthy participants under stress conditions or with a depressive or anxiety diagnosis: a meta-analysis of randomized controlled trials. Front Neurol. (2020) 11:421. doi: 10.3389/fneur.2020.00421, 32528399 PMC7257376

[27] Liu B He Y Wang M Liu J Ju Y Zhang Y . Efficacy of probiotics on anxiety—a meta-analysis of randomized controlled trials. Depress Anxiety. (2018) 35:935–45. doi: 10.1002/da.22811, 29995348

[28] Lew LC Hor YY Yusoff NAA Choi SB Yusoff MSB Roslan NS . Probiotic *Lactobacillus plantarum* P8 alleviated stress and anxiety while enhancing memory and cognition in stressed adults: a randomised, double-blind, placebo-controlled study. Clin Nutr. (2019) 38:2053–64. doi: 10.1016/j.clnu.2018.09.010, 30266270

[29] Zhang N Zhang Y Li M Wang W Liu Z Xi C . Efficacy of probiotics on stress in healthy volunteers: a systematic review and meta-analysis based on randomized controlled trials. Brain Behav. (2020) 10:e01699. doi: 10.1002/brb3.1699, 32662591 PMC7507034

[30] The World Bank. (2024). World Bank country and lending groups. Available online at: https://datahelpdesk.worldbank.org/knowledgebase/articles/906519-world-bank-country-and-lending-groups [Accessed September 9, 2025]

[31] Karl JP Hatch AM Arcidiacono SM Pearce SC Pantoja-Feliciano IG Doherty LA . Effects of psychological, environmental and physical stressors on the gut microbiota. Front Microbiol. (2018) 9:2013. doi: 10.3389/fmicb.2018.02013, 30258412 PMC6143810

[32] Cohen S Kamarck T Mermelstein R. A global measure of perceived stress. J Health Soc Behav. (1983) 24:385. doi: 10.2307/2136404, 6668417

[33] Wu S-I Wu C-C Cheng L-H Noble SW Liu C-J Lee Y-H . Psychobiotic supplementation of HK-PS23 improves anxiety in highly stressed clinical nurses: a double-blind randomized placebo-controlled study. Food Funct. (2022) 13:8907–19. doi: 10.1039/D2FO01156E, 35924970

[34] Hou K Wu Z-X Chen X-Y Wang J-Q Zhang D Xiao C . Microbiota in health and diseases. Signal Transduct Target Ther. (2022) 7:135. doi: 10.1038/s41392-022-00974-4, 35461318 PMC9034083

[35] Maher SE O’Brien EC Moore RL Byrne DF Geraghty AA Saldova R . The association between the maternal diet and the maternal and infant gut microbiome: a systematic review. Br J Nutr. (2023) 129:1491–9. doi: 10.1017/S0007114520000847, 32129734

[36] Karim MR Iqbal S Mohammad S Lee JH Jung D Mathiyalagan R . A review on impact of dietary interventions, drugs, and traditional herbal supplements on the gut microbiome. Microbiol Res. (2023) 271:127346. doi: 10.1016/j.micres.2023.127346, 36921399

[37] Almadi T Cathers I Hamdan Mansour AM Chow CM. An Arabic version of the perceived stress scale: translation and validation study. Int J Nurs Stud. (2012) 49:84–9. doi: 10.1016/j.ijnurstu.2011.07.012, 21851941

[38] Zubaidi A. M. Al-Saud N. H. Al-Qahtani X. A. Shaik S. A. Abdulla M. H. Al-Khayal K. A. . (2012). Bowel function and its associated variables in Saudi adults a population based study. Available online at: www.smj.org.sa (Accessed January 6, 2025). 22729117

[39] WHO (2020). The WHO STEPwise approach to noncommunicable disease risk factor surveillance. Available online at: https://cdn.who.int/media/docs/default-source/ncds/ncd-surveillance/steps/steps-manual.pdf?sfvrsn=c281673d_7 (Accessed January 6, 2025).

[40] Vickers AJ Altman DG. Statistics notes: Analysing controlled trials with baseline and follow up measurements. BMJ. (2001) 323:1123–4. doi: 10.1136/bmj.323.7321.1123, 11701584 PMC1121605

[41] Chahwan B Kwan S Isik A van Hemert S Burke C Roberts L. Gut feelings: a randomised, triple-blind, placebo-controlled trial of probiotics for depressive symptoms. J Affect Disord. (2019) 253:317–26. doi: 10.1016/j.jad.2019.04.097, 31078831

[42] Guan Y Zhu R Zhao W Wang L You L Zeng Z . Effects of Lacticaseibacillus paracasei K56 on perceived stress among pregraduate students: a double-blind, randomized, placebo-controlled trial. Front Nutr. (2025) 12:1544713. doi: 10.3389/fnut.2025.1544713, 40144570 PMC11936786

[43] Vambheim SM Daniali H Flaten MA. Placebo effects on stress, but not on pain reports. A multi-experiment study. Front Psychol. (2021) 12:639236. doi: 10.3389/fpsyg.2021.639236, 34163396 PMC8215114

[44] Fritz J Stochl J Kievit RA van Harmelen A-L Wilkinson PO. Tracking stress, mental health, and resilience factors in medical students before, during, and after a stress-inducing exam period: protocol and proof-of-principle analyses for the RESIST cohort study. JMIR Form Res. (2021) 5:e20128. doi: 10.2196/20128, 34100761 PMC8262546

[45] Amanvermez Y Zhao R Cuijpers P de Wit LM Ebert DD Kessler RC . Effects of self-guided stress management interventions in college students: a systematic review and meta-analysis. Internet Interv. (2022) 28:100503. doi: 10.1016/j.invent.2022.100503, 35242591 PMC8861419

[46] Bohlouli J Namjoo I Borzoo-Isfahani M Hojjati Kermani MA Balouch Zehi Z Moravejolahkami AR. Effect of probiotics on oxidative stress and inflammatory status in diabetic nephropathy: a systematic review and meta-analysis of clinical trials. Heliyon. (2021) 7:e05925. doi: 10.1016/j.heliyon.2021.e05925, 33490683 PMC7808957

[47] Dai Y Quan J Xiong L Luo Y Yi B. Probiotics improve renal function, glucose, lipids, inflammation and oxidative stress in diabetic kidney disease: a systematic review and meta-analysis. Ren Fail. (2022) 44:862–80. doi: 10.1080/0886022X.2022.2079522, 35611435 PMC9154786

[48] Den H Dong X Chen M Zou Z. Efficacy of probiotics on cognition, and biomarkers of inflammation and oxidative stress in adults with Alzheimer’s disease or mild cognitive impairment — a meta-analysis of randomized controlled trials. Aging. (2020) 12:4010–39. doi: 10.18632/aging.102810, 32062613 PMC7066922

[49] Krüger JF Hillesheim E Pereira ACSN Camargo CQ Rabito EI. Probiotics for dementia: a systematic review and meta-analysis of randomized controlled trials. Nutr Rev. (2021) 79:160–70. doi: 10.1093/nutrit/nuaa037, 32556236

[50] Vitellio P, Chira A, De Angelis M, Dumitrascu DL, Portincasa P. Probiotics in psychosocial stress and anxiety. A systematic review. J Gastrointest Liver Dis (2020) 29:77–83. doi:doi: 10.15403/jgld-352, 32176751

[51] Schmidt K Enge S Miller R. Reconsidering the construct validity of self-reported chronic stress: a multidimensional item response theory approach. Psychol Assess. (2020) 32:997–1014. doi: 10.1037/pas0000829, 32730073

[52] Binda S Tremblay A Iqbal UH Kassem O Le Barz M Thomas V . Psychobiotics and the microbiota–gut–brain Axis: where do we go from Here? Microorganisms. (2024) 12:634. doi: 10.3390/microorganisms12040634, 38674579 PMC11052108

[53] Rahmannia M Poudineh M Mirzaei R Aalipour MA Shahidi Bonjar AH Goudarzi M . Strain-specific effects of probiotics on depression and anxiety: a meta-analysis. Gut Pathog. (2024) 16:46. doi: 10.1186/s13099-024-00634-8, 39245752 PMC11382490

[54] Sanchez M Darimont C Panahi S Drapeau V Marette A Taylor V . Effects of a diet-based weight-reducing program with probiotic supplementation on satiety efficiency, eating behaviour traits, and psychosocial Behaviours in obese individuals. Nutrients. (2017) 9:284. doi: 10.3390/nu9030284, 28294985 PMC5372947

[55] Snigdha S Ha K Tsai P Dinan TG Bartos JD Shahid M. Probiotics: potential novel therapeutics for microbiota-gut-brain axis dysfunction across gender and lifespan. Pharmacol Ther. (2022) 231:107978. doi: 10.1016/j.pharmthera.2021.107978, 34492236

[56] Messaoudi M Lalonde R Violle N Javelot H Desor D Nejdi A . Assessment of psychotropic-like properties of a probiotic formulation (*Lactobacillus helveticus* R0052 and *Bifidobacterium longum* R0175) in rats and human subjects. Br J Nutr. (2011) 105:755–64. doi: 10.1017/S0007114510004319, 20974015

[57] Östlund-Lagerström L Kihlgren A Repsilber D Björkstén B Brummer RJ Schoultz I. Probiotic administration among free-living older adults: a double blinded, randomized, placebo-controlled clinical trial. Nutr J. (2015) 15:80. doi: 10.1186/s12937-016-0198-1, 27612653 PMC5018181

[58] Kudielka BM Kirschbaum C. Sex differences in HPA axis responses to stress: a review. Biol Psychol. (2005) 69:113–32. doi: 10.1016/j.biopsycho.2004.11.009, 15740829

[59] Govaert M Rotsaert C Vannieuwenhuyse C Duysburgh C Medlin S Marzorati M . Survival of probiotic bacterial cells in the upper gastrointestinal tract and the effect of the surviving population on the colonic microbial community activity and composition. Nutrients. (2024) 16:2791. doi: 10.3390/nu16162791, 39203927 PMC11357584

[60] Venema K Verhoeven J Verbruggen S Espinosa L Courau S. Probiotic survival during a multi-layered tablet development as tested in a dynamic, computer-controlled in vitro model of the stomach and small intestine (TIM-1). Lett Appl Microbiol. (2019) 69:325–32. doi: 10.1111/lam.13211, 31454425 PMC6856813

[61] Cook MT Tzortzis G Charalampopoulos D Khutoryanskiy VV. Microencapsulation of probiotics for gastrointestinal delivery. J Control Release. (2012) 162:56–67. doi: 10.1016/j.jconrel.2012.06.003, 22698940

[62] Tompkins T Mainville I Arcand Y. The impact of meals on a probiotic during transit through a model of the human upper gastrointestinal tract. Benefic Microbes. (2011) 2:295–303. doi: 10.3920/BM2011.0022, 22146689

[63] Treven P Paveljšek D Bogovič Matijašić B Mohar Lorbeg P. The effect of food matrix taken with probiotics on the survival of commercial probiotics in simulation of gastrointestinal digestion. Foods. (2024) 13:3135. doi: 10.3390/foods13193135, 39410170 PMC11475386

[64] Vasiliu O. The current state of research for psychobiotics use in the management of psychiatric disorders–a systematic literature review. Front Psychol. (2023) 14:1074736. doi: 10.3389/fpsyt.2023.1074736, 36911130 PMC9996157

[65] Hamidovic A Karapetyan K Serdarevic F Choi SH Eisenlohr-Moul T Pinna G. Higher circulating cortisol in the follicular vs. luteal phase of the menstrual cycle: a meta-analysis. Front Endocrinol (Lausanne). (2020) 11:311. doi: 10.3389/fendo.2020.00311, 32582024 PMC7280552

[66] Wastyk HC Fragiadakis GK Perelman D Dahan D Merrill BD Yu FB . Gut-microbiota-targeted diets modulate human immune status. Cell. (2021) 184:4137–4153.e14. doi: 10.1016/j.cell.2021.06.019, 34256014 PMC9020749

[67] Sejbuk M Siebieszuk A Witkowska AM. The role of gut microbiome in sleep quality and health: dietary strategies for microbiota support. Nutrients. (2024) 16:2259. doi: 10.3390/nu16142259, 39064702 PMC11279861

[68] Gualtieri P Marchetti M Cioccoloni G De Lorenzo A Romano L Cammarano A . Psychobiotics regulate the anxiety symptoms in carriers of allele a of IL-1 β gene: a randomized, placebo-controlled clinical trial. Mediat Inflamm. (2020) 2020:1–11. doi: 10.1155/2020/2346126, 32377159 PMC7199572

[69] Wang Y Wu Y Wang Y Xu H Mei X Yu D . Antioxidant properties of probiotic bacteria. Nutrients. (2017) 9:521. doi: 10.3390/nu9050521, 28534820 PMC5452251

[70] St-Amant A Bergdahl A. A systematic review and meta-analysis of randomized controlled trials investigating the effects of probiotics on oxidative stress in healthy adults. Clin Nutr ESPEN. (2023) 54:180–6. doi: 10.1016/j.clnesp.2023.01.016, 36963861

[71] Kato-Kataoka A Nishida K Takada M Suda K Kawai M Shimizu K . Fermented milk containing *Lactobacillus casei* strain Shirota prevents the onset of physical symptoms in medical students under academic examination stress. Benefic Microbes. (2016) 7:153–6. doi: 10.3920/BM2015.0100, 26689231

[72] Nishida K Sawada D Kuwano Y Tanaka H Rokutan K. Health benefits of *Lactobacillus gasseri* CP2305 tablets in young adults exposed to chronic stress: a randomized, double-blind, placebo-controlled study. Nutrients. (2019) 11:1859. doi: 10.3390/nu11081859, 31405122 PMC6723420

[73] Pinto-Sanchez MI Hall GB Ghajar K Nardelli A Bolino C Lau JT . Probiotic Bifidobacterium longum NCC3001 reduces depression scores and alters brain activity: a pilot study in patients with irritable bowel syndrome. Gastroenterology. (2017) 153:448–459.e8. doi: 10.1053/j.gastro.2017.05.003, 28483500

