# Supplementary material for: The effect of probiotic supplementation on perceived stress and bowel function in healthy young adults: evidence from a randomized controlled trial in Makkah
Source: Front Nutr. 2026 Jan 6;12:1717047. doi: 10.3389/fnut.2025.1717047 (PMC12815857; doi:10.3389/fnut.2025.1717047)
Supplement: Supplementary file 1 [file Table_1.docx]

Supplementary Material

Questionnaires presented first and second visit

**First visit Questionnaires**

**ID number رقم المشارك**

As a first step, body composition data were collected from the participants and documented within the questionnaires.

1. **InBody Questionnaire:**
   1. Age العمر
   2. Height الطول
   3. Weightالوزن
   4. BMIكتله الجسم
   5. Fat % نسبة الدهون
   6. Visceral fat
2. **Sociodemographic questions**
   1. Nationality الجنسية

- Saudi سعودي
- Other أخرى
  1. Where in Makkah do you live? في أي جهة من مكة تسكن
- North شمال
- South جنوب
- East شرق
- West غرب
  1. Educational level المستوى التعليمي
- Illiterate أّميّ
- Read and write يقرأ ويكتب
- Primary ابتدائي
- Intermediate متوسط
- High school ثانوي
- University جامعي
- Postgraduate (Master/PhD) دراسات عليا ماجستير أو دكتوراه
  1. Occupation الوظيفة
- Student طالب
- Unemployed غير موظف
- Military عسكري
- Clerical عمل مكتبي
- Field work عمل ميداني
- Teacher مدّرس
- Other أخرى
  1. I live in .. أقيم في
- Small apartmentشقه
- Large flat دور سكني
- Small house بيت شعبي
- Villa فيلا
- Refuse to answer امتنع عن الإجابة
- Other أخرى
  1. I practice physical activity /sports (you can choose more than one answer)  أمارس النشاطات البدنية/الرياضية (يمكن اختيار أكثر من إجابة)
- Daily يومي
- 3-4 days/week من 3 إلى 4 أيام في الأسبوع
- Weekly اسبوعي
- Monthly شهري
- No specific time ليس هناك وقت محدد
  1. Do you have any chronic illness? هل تعاني من أمراض مزمنة
- Liver diseases أمراض الكبد
- Thyroid disorders أمراض الغدة الدرقية
- Diabetes السكري
- Hypertension ارتفاع ضغط الدم
- Bowel/intestinal diseases التهابات الأمعاء
- I have no medical conditions لا أعاني من أي مرض
  1. Do you use any of the following medications? هل تستخدم أي من هذه الأدوية
- Insulin/diabetes regulatory الانسولين/ منظم السكر
- Laxatives ملينات/مسهلات
- Thyroid diseases medications أدوية الغدة الدرقية
- Hypertension medications أدوية ضغط الدم
- Inflammatory bowel disease أدوية التهابات الجهاز الهضمي
- I do not use any medications/لا أستخدم أي أدوية

1. **Bowel movement questionnaire**
   1. Do you think you have normal bowel movement? هل تعتقد أن عملية الإخراج لديك طبيعية

- Yes نعم
- No لا
  1. The amount of daily liquids مقدار السوائل التي أتناولها في اليوم
- One cup كوب واحد
- 2-4 cups ٢-٤ أكواب
- 4-6 cups ٤-٦ أكواب
- 6-8 cups ٦-٨ أكواب
- More than 8 cups أكثر من ٨ أكواب
  1. How many times do you defecate?
- Once/day أكثر من مره يومياً
- Once/week مره في الأسبوع
- More than once a week أكثر من مره في الأسبوع
- 3-7 times/week من ٣-٧ مرات في الأسبوع
  1. At any time during the day do you excrete?
- After getting out of bed                     بعد النهوض من النوم
- After breakfast بعد وجبة الإفطار
- After lunch بعد وجبة الغداء
- At night في الليل
- There is no specific time ليس هنالك وقت محدد

The questions between 3.5. to 3.9. afford more than one answer

**ملاحظة: يمكن اختيار أكثر من إجابة واحدة للأسئلة من 3.5 إلى 3.9.**

- 1. Adopted in the diet? أعتمد في غذائي على
- Fruits and vegetables الخضار والفواكه
- Meat and dairy products اللحوم ومنتجات الألبان
- Pepper and spices الفلفل والبهارات
- Rice and pasta الأرز والمعجنات
- Wheat and bran حبوب القمح والنخالة
  1. I suffer from difficulties during defecation. أعاني من صعوبات أثناء التبرز
- No لا
- Hurt اتألم
- Strainأحزق/أتزحر
- I notice there is blood الاحظ وجود دم
- I feel lumps during defecation أحس بزوائد تخرج أثناء التبرز
  1. I have problems after eating certain foods. أعاني من مشاكل بعد تناول أطعمة معينة
- No لا
- Pepper and spices الفلفل والبهارات
- Meat اللحوم
- Dairy products منتجات الألبان
- Rice and pasta الأرز والمعجنات
  1. These problems are? هذه المشاكل عبارة عن
- Nothing لا شيء
- Gas غازات
- Abdominal pain and discomfort الم في البطن وعدم ارتياح
- Hard stool صلابة وقساوة في البراز
- Diarrhea اسهال
- Soft stool براز لين
  1. I have a control problem?لدي مشكلة في التحكم
- No لا
- Gas بالغازات
- Soft stool البراز اللين
- Normal stool البراز العادي
- Urine بالبول
  1. I use laxative استخدام الملينات
- No لا
- Natural ملينات طبيعية
- Chemical ملينات كيميائية
  1. I notice may clothes get dirty as a result of inability to control my stool الاحظ اتساخ ملابسي لعدم مقدرتي على التحكم في اخراج البراز
- No لا
- Daily يومياً
- Weekly أسبوعياً
- Monthly شهرياً
  1. I had previously undergone surgery in intestine خضعت مسبقاً لعملية جراحية في الأمعاء
- No لا
- Small intestine في الأمعاء الدقيقة
- Colon في القولون
- Rectum في المستقيم
  1. I have undergone a surgical procedure on the anal area خضعت لعملية جراحية في فتحة الشرج
- No لا
- Congenital anomaly عيوب ولادية وخلقية
- Hemorrhoid البواسير
- Anal fistula الناسور الشرجي

1. Probiotic awareness Questionnaire
   1. Do you consume probiotics **هل تتعاطى بكتيريا البروبيوتيك النافعة؟**

- Yes **نعم**
- No **لا**
  1. How do you select which probiotics to consume? **كيف تختار أي نوع من بكتيريا البروبيوتيك النافعة لتعاطيها؟**
- Depending on the number of bacteria بحسب عدد البكتيريا
- Depending on the price بحسب السعر
- Depending on the company بحسب الشركة
- I don’t care لا أهتم
- I don’t know لا أعلم
  1. When do you consume them? **متى تتعاطاها؟**
- Before meal قبل الوجبات
- With meal مع الوجبات
- Within 30 minutes following a mealخلال نصف ساعة بعد الوجبات
- After a meal بعد الوجبات
- I don’t know لا أعلم
  1. How often do consume them**? كم مرة تتعاطاها؟**
- Once a day مرة في اليوم
- Once in 2 days مرة في اليومين
- Once a week مرة في الأسبوع
- Do not follow any pattern **بدون نظام معين**
- As and when required عند اللزوم
- I don’t know لا أعلم
  1. Were probiotics beneficial to you? **هل استفدت من تعاطي بكتيريا البروبيوتيك النافعة؟**
- Yes نعم
- No لا
- I don’t know لا أعلم
  1. Will you consume them in the future? **هل ستتعاطاها في المستقبل؟**
- Yes نعم
- No لا
- I don’t know لا أعلم
  1. Do you recommend others to take probiotics**? هل تنصح الآخرين بتعاطيها؟**
- Yes نعم
- No لا
- I don’t know لا أعلم

1. **Stress questionnaire**

1-In the last month, how often have you been upset because of something that happened unexpectedly?

خلال الشهر الماضي، إلى أي مدى أحسست بالانزعاج بسبب حدوث أمر غير متوقع؟

0=Never اطلاقاً (أبداً)

1=Rarely نادراً

2=Sometime احياناً

3=Often كثيراً

4= Always دائماً (في أغلب الأحيان)

2-In the last month, how often have you felt that you were unable to control the important things in your life?

خلال الشهر الماضي، إلى أي مدى أحسست بعدم القدرة على التحكم في الأمور الهامة بحياتك ؟

0=Never اطلاقاً (أبداً)

1=Rarely نادراً

2=Sometime احياناً

3=Often كثيراً

4= Always دائماً (في أغلب الأحيان)

3-In the last month, how often have you felt nervous and stressed?

خلال الشهر الماضي، إلى أي مدى أحسست بالتوتر والضغط النفسي ؟

0=Never اطلاقاً (أبداً)

1=Rarely نادراً

2=Sometime احياناً

3=Often كثيراً

4= Always دائماً (في أغلب الأحيان)

4-In the last  month, how often have you felt confident about your ability to handle your personal problems?

خلال الشهر الماضي، إلى أي مدى أحسست بالثقة في قدرتك على التعامل مع مشاكلك الخاصة ؟

4=Never اطلاقاً (أبداً)

3=Rarely نادراً

2=Sometime احياناً

1=Often كثيراً

0= Always دائماً (في أغلب الأحيان)

5-In the last month, how often have you felt that things were going your way?

خلال الشهر الماضي، إلى أي مدى أحسست أن الأمور تسير كما تريد ؟

4=Never اطلاقاً (أبداً)

3=Rarely نادراً

2=Sometime احياناً

1=Often كثيراً

0= Always دائماً (في أغلب الأحيان)

6-In the last month, how often have you found that you could not cope with all the things that you had to do?

خلال الشهر الماضي، إلى أي مدى وجدت نفسك غير قادر على التأقلم مع كل الأمور الواجب عليك القيام بها ؟

0=Never اطلاقاً (أبداً)

1=Rarely نادراً

2=Sometime احياناً

3=Often كثيراً

4= Always دائماً (في أغلب الأحيان)

7-In the last month, how often have you been able to control irritations in your life?

خلال الشهر الماضي، إلى أي مدى تمكنت من التحكم في الأمور التي تزعجك ؟

4=Never اطلاقاً (أبداً)

3=Rarely نادراً

2=Sometime احياناً

1=Often كثيراً

0= Always دائماً (في أغلب الأحيان)

8-In the last month, how often have you felt that you were on top of things?

خلال الشهر الماضي، إلى أي مدى أحسست بأنك تمتلك زمام الأمور ( مسيطر على كافة امورك ) ؟

4=Never اطلاقاً (أبداً)

3=Rarely نادراً

2=Sometime احياناً

1=Often كثيراً

0= Always دائماً (في أغلب الأحيان)

9-In the last month, how often have you been angered because of things that happened that were outside of your control?

خلال الشهر الماضي، إلى أي مدى أحسست بالغضب بسبب أمور خارجة عن تحكمك؟

0=Never اطلاقاً (أبداً)

1=Rarely نادراً

2=Sometime احياناً

3=Often كثيراً

4= Always دائماً (في أغلب الأحيان)

10-In the last month, how often have you felt difficulties were piling up so high that you could not overcome them?

خلال الشهر الماضي، إلى أي مدى أحسست بأن الصعاب تتراكم عليك لدرجة انك لم تعد تستطيع التغلب عليها ؟

0=Never اطلاقاً (أبداً)

1=Rarely نادراً

2=Sometime احياناً

3=Often كثيراً

4= Always دائماً (في أغلب الأحيان)

**Second Visit Questionnaires**

**ID number رقم المشارك**

1. **InBody Questionnaire:**
   1. Age العمر
   2. Height الطول
   3. Weightالوزن
   4. BMIكتله الجسم
   5. Fat % نسبة الدهون
   6. Visceral fat
   7. I practice physical activity /sports (you can choose more than one answer)  أمارس النشاطات البدنية/الرياضية (يمكن اختيار أكثر من إجابة)

- Daily يومي
- 3-4 days/week من 3 إلى 4 أيام في الأسبوع
- Weekly اسبوعي
- Monthly شهري
- No specific time ليس هناك وقت محدد
  1. Do you have any chronic illness? هل تعاني من أمراض مزمنة
- Liver diseases أمراض الكبد
- Thyroid disorders أمراض الغدة الدرقية
- Diabetes السكري
- Hypertension ارتفاع ضغط الدم
- Bowel/intestinal diseases التهابات الأمعاء
- I have no medical conditions لا أعاني من أي مرض
  1. Do you use any of the following medications? هل تستخدم أي من هذه الأدوية
- Insulin/diabetes regulatory الانسولين/ منظم السكر
- Laxatives ملينات/مسهلات
- Thyroid diseases medications أدوية الغدة الدرقية
- Hypertension medications أدوية ضغط الدم
- Inflammatory bowel disease أدوية التهابات الجهاز الهضمي
- I do not use any medications/لا أستخدم أي أدوية

1. **Bowel movement questionnaire**
   1. Do you think you have normal bowel movement? هل تعتقد أن عملية الإخراج لديك طبيعية

- Yes نعم
- No لا
  1. The amount of daily liquids مقدار السوائل التي أتناولها في اليوم
- One cup كوب واحد
- 2-4 cups ٢-٤ أكواب
- 4-6 cups ٤-٦ أكواب
- 6-8 cups ٦-٨ أكواب
- More than 8 cups أكثر من ٨ أكواب
  1. How many times do you defecate?
- Once/day أكثر من مره يومياً
- Once/week مره في الأسبوع
- More than once a week أكثر من مره في الأسبوع
- 3-7 times/week من ٣-٧ مرات في الأسبوع
  1. At any time during the day do you excrete?
- After getting out of bed                     بعد النهوض من النوم
- After breakfast بعد وجبة الإفطار
- After lunch بعد وجبة الغداء
- At night في الليل
- There is no specific time ليس هنالك وقت محدد

The questions between 3.5. to 3.9. afford more than one answer

**ملاحظة: يمكن اختيار أكثر من إجابة واحدة للأسئلة من 3.5 إلى 3.9.**

- 1. Adopted in the diet? أعتمد في غذائي على
- Fruits and vegetables الخضار والفواكه
- Meat and dairy products اللحوم ومنتجات الألبان
- Pepper and spices الفلفل والبهارات
- Rice and pasta الأرز والمعجنات
- Wheat and bran حبوب القمح والنخالة
  1. I suffer from difficulties during defecation. أعاني من صعوبات أثناء التبرز
- No لا
- Hurt اتألم
- Strainأحزق/أتزحر
- I notice there is blood الاحظ وجود دم
- I feel lumps during defecation أحس بزوائد تخرج أثناء التبرز
  1. I have problems after eating certain foods. أعاني من مشاكل بعد تناول أطعمة معينة
- No لا
- Pepper and spices الفلفل والبهارات
- Meat اللحوم
- Dairy products منتجات الألبان
- Rice and pasta الأرز والمعجنات
  1. These problems are? هذه المشاكل عبارة عن
- Nothing لا شيء
- Gas غازات
- Abdominal pain and discomfort الم في البطن وعدم ارتياح
- Hard stool صلابة وقساوة في البراز
- Diarrhea اسهال
- Soft stool براز لين
  1. I have a control problem?لدي مشكلة في التحكم
- No لا
- Gas بالغازات
- Soft stool البراز اللين
- Normal stool البراز العادي
- Urine بالبول
  1. I use laxative استخدام الملينات
- No لا
- Natural ملينات طبيعية
- Chemical ملينات كيميائية
  1. I notice may clothes get dirty as a result of inability to control my stool الاحظ اتساخ ملابسي لعدم مقدرتي على التحكم في اخراج البراز
- No لا
- Daily يومياً
- Weekly أسبوعياً
- Monthly شهرياً
  1. I had previously undergone surgery in intestine خضعت مسبقاً لعملية جراحية في الأمعاء
- No لا
- Small intestine في الأمعاء الدقيقة
- Colon في القولون
- Rectum في المستقيم
  1. I have undergone a surgical procedure on the anal area خضعت لعملية جراحية في فتحة الشرج
- No لا
- Congenital anomaly عيوب ولادية وخلقية
- Hemorrhoid البواسير
- Anal fistula الناسور الشرجي

1. **Stress questionnaire.**

1-In the last month, how often have you been upset because of something that happened unexpectedly?

خلال الشهر الماضي، إلى أي مدى أحسست بالانزعاج بسبب حدوث أمر غير متوقع؟

0=Never اطلاقاً (أبداً)

1=Rarely نادراً

2=Sometime احياناً

3=Often كثيراً

4= Always دائماً (في أغلب الأحيان)

2-In the last month, how often have you felt that you were unable to control the important things in your life?

خلال الشهر الماضي، إلى أي مدى أحسست بعدم القدرة على التحكم في الأمور الهامة بحياتك ؟

0=Never اطلاقاً (أبداً)

1=Rarely نادراً

2=Sometime احياناً

3=Often كثيراً

4= Always دائماً (في أغلب الأحيان)

3-In the last month, how often have you felt nervous and stressed?

خلال الشهر الماضي، إلى أي مدى أحسست بالتوتر والضغط النفسي ؟

0=Never اطلاقاً (أبداً)

1=Rarely نادراً

2=Sometime احياناً

3=Often كثيراً

4= Always دائماً (في أغلب الأحيان)

4-In the last  month, how often have you felt confident about your ability to handle your personal problems?

خلال الشهر الماضي، إلى أي مدى أحسست بالثقة في قدرتك على التعامل مع مشاكلك الخاصة ؟

4=Never اطلاقاً (أبداً)

3=Rarely نادراً

2=Sometime احياناً

1=Often كثيراً

0= Always دائماً (في أغلب الأحيان)

5-In the last month, how often have you felt that things were going your way?

خلال الشهر الماضي، إلى أي مدى أحسست أن الأمور تسير كما تريد ؟

4=Never اطلاقاً (أبداً)

3=Rarely نادراً

2=Sometime احياناً

1=Often كثيراً

0= Always دائماً (في أغلب الأحيان)

6-In the last month, how often have you found that you could not cope with all the things that you had to do?

خلال الشهر الماضي، إلى أي مدى وجدت نفسك غير قادر على التأقلم مع كل الأمور الواجب عليك القيام بها ؟

0=Never اطلاقاً (أبداً)

1=Rarely نادراً

2=Sometime احياناً

3=Often كثيراً

4= Always دائماً (في أغلب الأحيان)

7-In the last month, how often have you been able to control irritations in your life?

خلال الشهر الماضي، إلى أي مدى تمكنت من التحكم في الأمور التي تزعجك ؟

4=Never اطلاقاً (أبداً)

3=Rarely نادراً

2=Sometime احياناً

1=Often كثيراً

0= Always دائماً (في أغلب الأحيان)

8-In the last month, how often have you felt that you were on top of things?

خلال الشهر الماضي، إلى أي مدى أحسست بأنك تمتلك زمام الأمور ( مسيطر على كافة امورك ) ؟

4=Never اطلاقاً (أبداً)

3=Rarely نادراً

2=Sometime احياناً

1=Often كثيراً

0= Always دائماً (في أغلب الأحيان)

9-In the last month, how often have you been angered because of things that happened that were outside of your control?

خلال الشهر الماضي، إلى أي مدى أحسست بالغضب بسبب أمور خارجة عن تحكمك؟

0=Never اطلاقاً (أبداً)

1=Rarely نادراً

2=Sometime احياناً

3=Often كثيراً

4= Always دائماً (في أغلب الأحيان)

10-In the last month, how often have you felt difficulties were piling up so high that you could not overcome them?

خلال الشهر الماضي، إلى أي مدى أحسست بأن الصعاب تتراكم عليك لدرجة انك لم تعد تستطيع التغلب عليها ؟

0=Never اطلاقاً (أبداً)

1=Rarely نادراً

2=Sometime احياناً

3=Often كثيراً

4= Always دائماً (في أغلب الأحيان)

Figure S1. Methodology and study design.


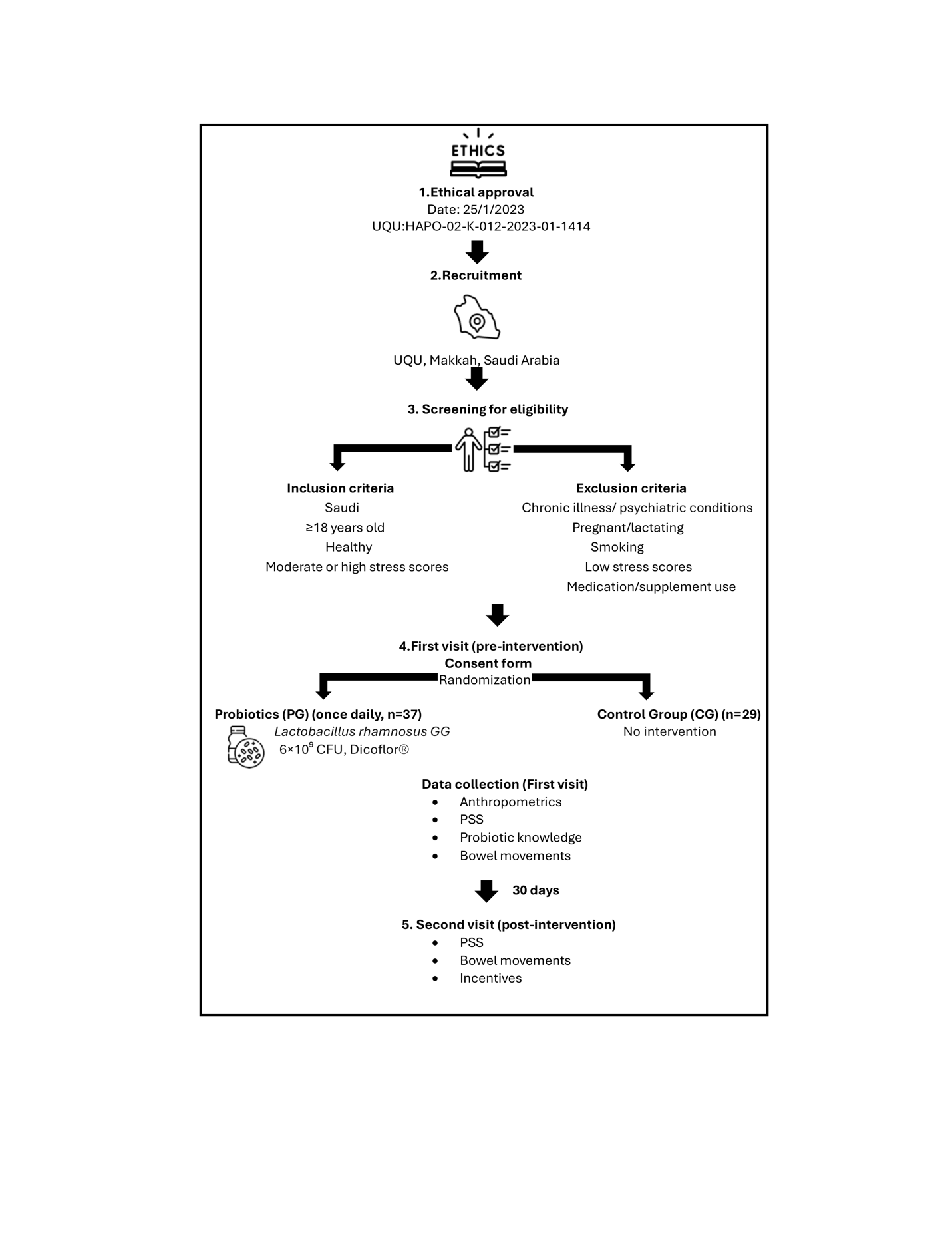


Table S1. Logistic Regression Analysis of Bowel Movement Characteristics at Baseline in the Probiotic and Control Groups

| **Variables** | **OR [95%CI)**  **Probiotic vs. Control** |
| --- | --- |
| **Do you think you have a normal bowel movement?** |  |
| Yes | 0.359 [0.09, 1.47] |
| No | Reference |
| **The amount of daily liquids** |  |
| 1–4 cups | Reference |
| 4–6 cups | 0.239*[0.07,0.83] |
| >6 cups | 0.667 [ 0.20, 2.26] |
| **Frequency of defecation** |  |
| Once/day | Reference |
| Once/week | 0.410[0.3, 4.88] |
| More than once a week | 0.818 [0.18, 3.74] |
| 3–7 times/week | 1.64 [0.47, 5.66] |
| **I have problems after eating certain foods** |  |
| No | Reference |
| Yes | 1.299[0.49, 3.45] |
| **These problems are** |  |
| No issues | Reference |
| Gas/Diarrhea/Pain | 0.824 [ 0.27, 2.54] |
| More than one answer | 1.647 [ 0.46, 5.96] |
| **I have a control problem** |  |
| No | Reference |
| Yes | 0.977 [ 0.24, 4.02] |
| **Difficulties during defecation** |  |
| No | Reference |
| Yes | 1.778 [0.53, 5.94] |

Data is presented as OR (95% CI); logistic regression was used.

Table S2. Logistic Regression Analysis of Bowel Movement Characteristics Post-intervention in the Probiotic and Control Groups

| **Variables** | **OR (95%CI)**  **Probiotic vs. Control** |
| --- | --- |
| **Do you think you have a normal bowel movement?** |  |
| Yes | 1.308 [0.24, 7.02] |
| No | Reference |
| **The amount of daily liquids** |  |
| 1–4 cups | Reference |
| 4–6 cups | 0.206*[0.06,0.77] |
| >6 cups | 0.576 [ 0.17, 1.91] |
| **Frequency of defecation** |  |
| Once/day | Reference |
| Once/week | 1.385[0.21, 9.24] |
| More than once a week | 0.692 [0.20, 2.43] |
| 3–7 times/week | 0.846 [0.25, 2.88] |
| **I have problems after eating certain foods** |  |
| No | Reference |
| Yes | 0.580[0.22, 1.57] |
| **These problems are** |  |
| No issues | Reference |
| Gas/Diarrhea/Pain | 0.462 [ 0.13, 1.64] |
| More than one answer | 0.337 [ 0.09, 1.23] |
| **I have a control problem** |  |
| No | Reference |
| Yes | 1.191 [ 0.19, 7.65] |
| **Difficulties during defecation** |  |
| No | Reference |
| Yes | 0.645 [0.77, 3.46] |

Data is presented as OR (95% CI); logistic regression was used.
